# Tracking Lysosome Migration within Chinese Hamster Ovary (CHO) Cells Following Exposure to Nanosecond Pulsed Electric Fields

**DOI:** 10.3390/bioengineering5040103

**Published:** 2018-11-23

**Authors:** Gary L. Thompson, Hope T. Beier, Bennett L. Ibey

**Affiliations:** 1 Department of Chemical Engineering, Rowan University, Glassboro, NJ 08028, USA; 2 Human Effectiveness Directorate, 711th Human Performance Wing, Air Force Research Laboratory, Joint Base San Antonio—Fort Sam Houston, San Antonio, TX 78234, USA; hopebeier@gmail.com (H.T.B.); bennettibey@gmail.com (B.L.I.)

**Keywords:** nsPEF, nanopores, exocytosis, biomembrane, calcium

## Abstract

Above a threshold electric field strength, 600 ns-duration pulsed electric field (nsPEF) exposure substantially porates and permeabilizes cellular plasma membranes in aqueous solution to many small ions. Repetitive exposures increase permeabilization to calcium ions (Ca^2+^) in a dosage-dependent manner. Such exposure conditions can create relatively long-lived pores that reseal after passive lateral diffusion of lipids should have closed the pores. One explanation for eventual pore resealing is active membrane repair, and an ubiquitous repair mechanism in mammalian cells is lysosome exocytosis. A previous study shows that intracellular lysosome movement halts upon a 16.2 kV/cm, 600-ns PEF exposure of a single train of 20 pulses at 5 Hz. In that study, lysosome stagnation qualitatively correlates with the presence of Ca^2+^ in the extracellular solution and with microtubule collapse. The present study tests the hypothesis that limitation of nsPEF-induced Ca^2+^ influx and colloid osmotic cell swelling permits unabated lysosome translocation in exposed cells. The results indicate that the efforts used herein to preclude Ca^2+^ influx and colloid osmotic swelling following nsPEF exposure did not prevent attenuation of lysosome translocation. Intracellular lysosome movement is inhibited by nsPEF exposure(s) in the presence of PEG 300-containing solution or by 20 pulses of nsPEF in the presence of extracellular calcium. The only cases with no significant decreases in lysosome movement are the sham and exposure to a single nsPEF in Ca^2+^-free solution.

## 1. Introduction

Exposure to nanosecond pulsed electric fields (nsPEF) creates nanopores within lipid bilayer membranes of cells. One hypothesized mechanism of pore formation that is supported by molecular dynamics simulations is that penetration of interfacial water molecules into the hydrophobic core of the bilayer increases with localized electric field strength [1]. Whether the pore is irreversible or reversible, and its lifetime, correlates with pulse duration, localized electric field magnitude, and exposure dosage. Earlier theoretical studies predict that for a typical, spherical cell with a 10-μm diameter, pulse durations greater than 100 ns at 13.3 kV/cm exceed the plasma membrane charging time, reaching the critical transmembrane voltage of 1 V to induce electroporation [2,3], and pores form with the highest density facing the anode. Given exposure to a 600-ns PEF at 16.2 kV/cm, the entire plasma membrane becomes thoroughly porated [4], and pore lifetimes are on the order of minutes, which extends beyond the timeframe for resealing via passive lateral diffusion of lipids [5].

Living mammalian cells possess a host of active mechanisms for repairing plasma membrane damage, including lysosomal exocytosis as a restoration mechanism. Disruption of the plasma membrane allows for an influx of extracellular Ca^2+^ that activates membrane repair machinery [6]. Briefly, microtubule-associated motor proteins transport lysosomes to the damage site, where the localized [Ca^2+^] accelerates fusion of accumulated lysosomes with the plasma membrane. Previously, we qualitatively observed that nsPEF exposure in a Ca^2+^-containing solution can disrupt cytoskeletal structure and consequently halt lysosomal movement. Here, nsPEF-induced interruption of lysosomal translocation is quantified using a mean square displacement (MSD) analysis [7], confirming observations that decreases in the transport of lysosomes occur when microtubules are constrained or likely disrupted.

## 2. Materials and Methods

### 2.1. Cell Culture Procedures

Adherent Chinese hamster ovary (CHO-K1, ATCC, Manassas, VA, #CCL-61) cells have been established as appropriate cellular models for bioelectrochemical studies because of their dearth of excitable channels and relative ease of culturing and transfection [8]. For visualization of lysosomes, CHO cells were transfected with red fluorescent protein–lysosomal-associated membrane protein 1 (RFP-Lamp1) using an Effectene kit (Qiagen, Gaithersburg, MD, USA, #301425) and maintained with G418 (Calbiochem #345812). Cells were grown in 75-cm^2^ flasks at 37 °C with 5% CO_2_ in air. Ham’s F12K medium supplemented with 10% fetal bovine serum and 1% penicillin/streptomycin was used for cell propagation. For imaging, cells were detached from flasks using trypsin/EDTA (ATCC, #30-2101) and were incubated in full-serum medium on poly-L-lysine (PLL)-coated, 10-mm-diameter, glass-bottom dishes (MatTek, Ashland, MA, USA) for 16 h.

Microscopy imaging and nsPEF exposures were performed within a custom buffer solution made of 2 mM MgCl_2_, 5 mM KCl, 10 mM 4-(2-hydroxyethyl)-1-piperazinee-thanesulfonic acid (HEPES), 10 mM Glucose, 2 mM CaCl_2_, and 135 mM NaCl (Sigma-Aldrich, St. Louis, MO, USA). The buffer osmolarity was measured and pH was adjusted to 7.4 using NaOH. In experiments where calcium was removed from the outside solution, 2 mM CaCl_2_ was replaced by 2 mM potassium ethylene glycol tetraacetic acid (K-EGTA) (Sigma-Aldrich) added to the buffer solution. Custom buffer solutions containing polyethylene glycol (PEG, number average molecular weight M_n_ = 300, Sigma-Aldrich, #202371) were prepared as by Nesin et al. [9]. In short, 100 mM NaCl in the Ca^2+^-depleted custom buffer was replaced with 152 mM PEG, so that only 35 mM NaCl was added. Osmolality of the final solution was confirmed to be between 290 and 300 mOsm with a freezing point osmometer (Advanced Instruments, Inc., Norwood, MA, USA, Model 3250). Imaging was performed at room temperature, between 22 °C and 24 °C.

### 2.2. Nanosecond Pulse Generator and Exposure Setup

Exposures were delivered via a custom-crafted microelectrode composed of two parallel tungsten wires (100-μm separation) and mounted at a 30–35° angle on an electronic micromanipulator (Sutter Instrument, Novato, CA, USA, MP-225). The microelectrode was positioned 50 μm above the coverslip surface for better uniformity of nsPEF exposure. The electric field amplitude delivered at the cell position for this configuration has been calculated using a finite-difference time-domain (FDTD) model in a previous publication [10]. Supplying 999 V, an electric field magnitude of 16.2 kV/cm at the imaged cell was predicted. For pulse durations of 600 ns, representative oscilloscope traces demonstrating pulse shapes, durations, and applied voltages delivered by this same system in each solution tested were published in Figure 1 of Thompson et al. [11], and a representative trace is shown in Supplemental Figure S1. Similar nsPEF exposure systems also were described previously [5,12], and a schematic diagram of the exact same nsPEF exposure system used herein was provided by Roth et al. [12].

### 2.3. Confocal Fluorescence Imaging

CHO cells transfected with RFP-Lamp1 were imaged using a Zeiss LSM 710 (Thornwood, NY, USA) laser scanning confocal microscope with a DIC 40×, 1.2 NA oil objective. Immediately upon nsPEF exposure, confocal images of a 2-μm slice in the approximate center of the cells were acquired either every 1 s for 480 s for solutions containing PEG, or every 3 s for 360 s for other solutions. Exposure to nsPEF occurred half-way through acquisition of an image sequence, i.e., between 240 s (‘Before’) and 241 s (‘After’) for solutions containing PEG, and between 180 s (‘Before’) and 181 s (‘After’) for other solutions. For comparison of nsPEF-exposure-induced effects in PEG-containing solutions and other solutions, only the middle 360 s were analyzed and reported for PEG-containing solutions. Three minutes was reported by Reddy et al. [13] to be an adequate time for measurable lysosome exocytosis to occur in response to plasma membrane ‘wounding’ and subsequent elevation of intracellular concentration of Ca^2+^. This three-minute period following nsPEF exposure also took into consideration the preclusion of cell swelling in PEG 300-containing solution during the initial five minutes following nsPEF exposure, as reported by Nesin et al. [9] and Thompson et al. [11].

### 2.4. Image Processing and Lysosome Tracking

Images were post-processed for single particle tracking (SPT) using Fiji TrackMate (version 2.2.0) [14]. Images were split into separate channels, and the fluorescence channel was kept for subsequent analyses. Background subtraction was performed using a rolling ball of five pixels. All image slices were then despeckled once. Contrast was enhanced with saturated pixels at 0.4%, and the histogram was normalized for all slices. Individual cell boundaries (i.e., the region of interest) were selected and pixel dimensions confirmed before proceeding with particle tracking.

To automatically detect lysosomes, TrackMate’s Laplacian of Gaussian (LoG) segmentation detector was selected, and an estimated particle diameter of 1.0 μm was set, with a threshold of 0.05, no median filter, and subpixel localization enabled. Initial thresholding of the number of spots to be analyzed was based on the Quality value determined by TrackMate’s segmentation algorithm. Spots with poor Quality values deviated strongly from estimated particle parameters and typically aggregated into a ‘first’ histogram peak. Therefore, initial spot thresholding was set at the ‘second’ peak, or if there was no second peak, the number of spots to be analyzed was restricted to less than 5000 to prevent computational performance problems. The HyperStack Displayer then was used to observe spots. Following initial filtering and display, spots were filtered further by mean intensity (~10) to include all visually identified lysosomes. To perform SPT, the Simple Linear Assignment Problem (LAP) Tracker was used with a linking maximum distance of 1.94 μm, inclusion of gaps up to 1.34 μm over six frames, and no merging. Tracks were filtered to include at least five spots, to account for all observable movement of lysosomes.

### 2.5. Data Analysis

Analysis of tracks was accomplished using the MATLAB (MathWorks, Natick, MA, R2014a) class “Mean square displacement (MSD) analysis of particle trajectories (i.e., @msdanalyzer, modified from version 1.2.0.0)” by Jean-Yves Tinevez [15] (https://github.com/tinevez). Since lysosome translocation following plasma membrane damage depends upon molecular motors associated with the microtubule network, a nonlinear MSD analysis was used to model the directed motion [7,16]. Each MSD curve was fit using the following polynomial expression for MSD (〈r^2^〉) as a function of time (t) that includes two coefficients representing the diffusion coefficient (D) and the velocity of directed motion (V).

〈r^2^〉 = 4Dt + V^2^t^2^. (1)

Curves that displayed a goodness of fit less than 80% were omitted from further analyses.

Nonparametric statistical tests were used to compare data samples from before and after nsPEF exposure. The Wilcoxon rank-sum test (i.e., the Mann–Whitney U-test) was performed in Matlab on the distributions of the integral number of lysosome tracks, including at least five consecutive spot detections. The two-sample Kolmogorov–Smirnov (K-S) test was performed in Excel using the add-in Real Statistics Resource Pack [17] on the distributions of the diffusion coefficients and particle velocities, which were assumed to be sampled from a population with a continuous, cumulative distribution.

Whereas diffusion coefficients were derived from the nonlinear MSD curve fits, the statistically tested resultant velocity values were not correlated with track analyses and instead were calculated from frame-to-frame measurements of paired displacements along the x- and y-axes. Therefore, the distributions of resultant lysosome velocities did not distinguish between retrograde and anterograde translocations, and the K-S test was used to find any difference between the distributions before and after exposure. Comparison between conditions using cell-to-cell mean values was accomplished using the two-tailed Student’s *t*-test in Microsoft Excel for Mac (Version 16.14.1).

## 3. Results

### 3.1. The Number of Lysosome Tracks Decreases Following nsPEF Exposure

**Figure 1:**
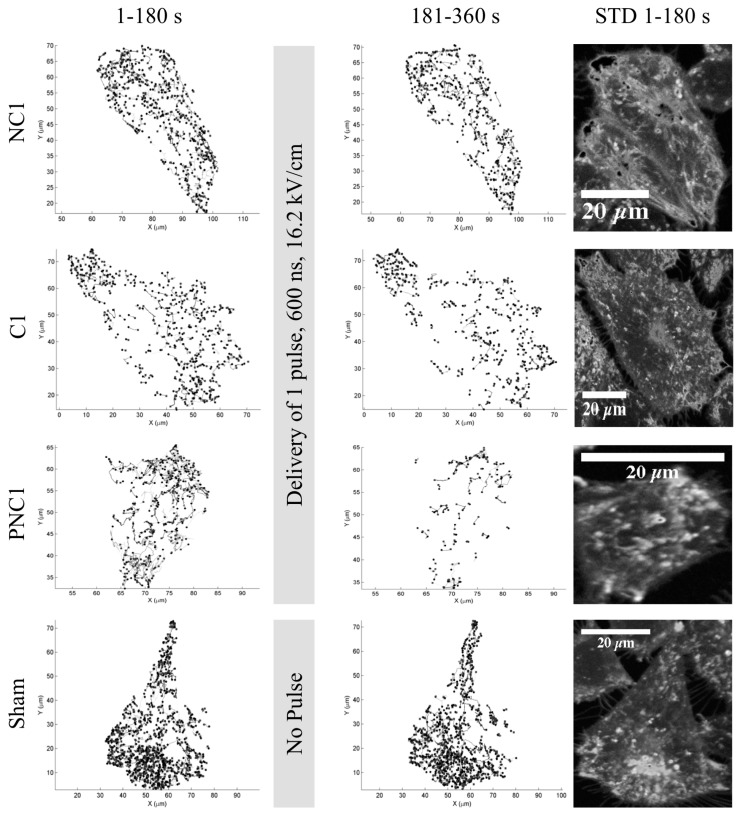
Tracks of lysosome movement show decreased numbers and lengths of lysosome trajectories for cells before (**left**) and after (**right**) exposure to a single, 600-ns pulsed electric field (PEF) at 16.2 kV/cm in solutions without external Ca^2+^ (NC1), with external Ca^2+^ (C1), or with PEG and without Ca^2+^ (PNC1). A sham exposure (i.e., a nanosecond PEF (nsPEF) was not delivered) in PNC is represented on the bottom. The standard deviation of a stack of representative confocal fluorescence images of the cells are shown on the far right.

Representative images of particle tracks within cells before and after nsPEF exposure are shown in Figure 1, along with the standard deviation image of all frames before exposure from the fluorescence channel corresponding to lysosomes. Decreases in the number of lysosome tracks are apparent in the presence of extracellular Ca^2+^ or PEG. Enlarged images of these particle tracks are given in Figures S2–S5. Also, the solution containing both PEG and extracellular Ca^2+^ (PC) has been tested; however, there is only one image sequence for each number of nsPEF exposure(s), i.e., 1 and 20 pulses. Since statistical analyses are not possible, preliminary results for this condition are provided in the Supplemental Materials. Figure S6 shows PC 1 particle tracks. Counting tracks before and after exposure confirms the trend seen in the representative images of Figure 1.

**Figure 2:**
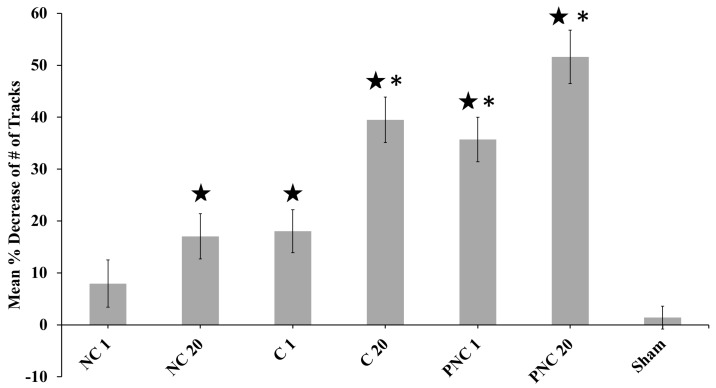
The mean percent decreases in the number of tracks exhibiting directed motion per cell, following nsPEF delivery, reveal that all exposures result in decreased tracks. Of the conditions tested, only the sham and the single nsPEF exposure in Ca^2+^-free solution (NC 1) do not have a significant decrease in the number of tracks (that contain appropriately sized particles detected in at least five consecutive frames). Error bars represent the cell-to-cell standard error of the mean (SEM), and star shapes (★) denote statistical significance using the Rank-Sum (rs) test at alpha = 0.01 compared to pre-exposure. Asterisks (*) denote statistical significance using a Student’s two-tailed *t*-test (tt) with alpha = 0.01 compared to sham. Values plotted in the graph are: NC 1 (number of cells (n) = 6, Y = 7.95%, SEM = 4.54, rs-p = 0.240, tt p = 0.234); NC 20 (n = 5, Y = 17.0%, SEM = 4.37, rs p = 0.00866; tt p = 0.0185); C 1 (n = 3, Y = 18.1%, SEM = 4.14, rs p = 0.0238, tt p = 0.0378); C 20 (n = 9, Y = 39.5%, SEM = 4.38, rs p = 0.000400, tt p = 8.43 × 10^−6^); PNC 1 (n = 22, Y = 35.7%, SEM = 4.38, rs p = 0.000466, tt p = 1.38 × 10^−7^); PNC 20 (n = 7, Y = 51.6%, SEM = 5.17, rs p = 0.00117, tt p = 1.93 × 10^−5^); and sham (n = 6, Y = 1.39%, SEM = 2.18, rs p = 1.00, tt p = 1.00).

Graphing the mean percent decrease of the number of tracks per cell reveals that only the sham condition and a single nsPEF exposure in extracellular solution without Ca^2+^ do not have a statistically significant decrease in the number of tracks, using the Wilcoxon rank-sum test at alpha = 0.05 (Figure 2). Exposures with 20 pulses, extracellular Ca^2+^, or solution containing PEG exhibit a statistically significant decrease in the number of tracks compared to before exposure. According to the two-tailed Student’s *t*-test at alpha = 0.01, these decreases are significantly different from the sham condition for 20 pulses in solution without Ca^2+^, and either 1 or 20 pulses in the presence of extracellular Ca^2+^ or PEG.

### 3.2. Exposure to nsPEF Decreases MSD, Diffusion Coefficients, and Velocities of Lysosome Translocation

**Figure 3:**
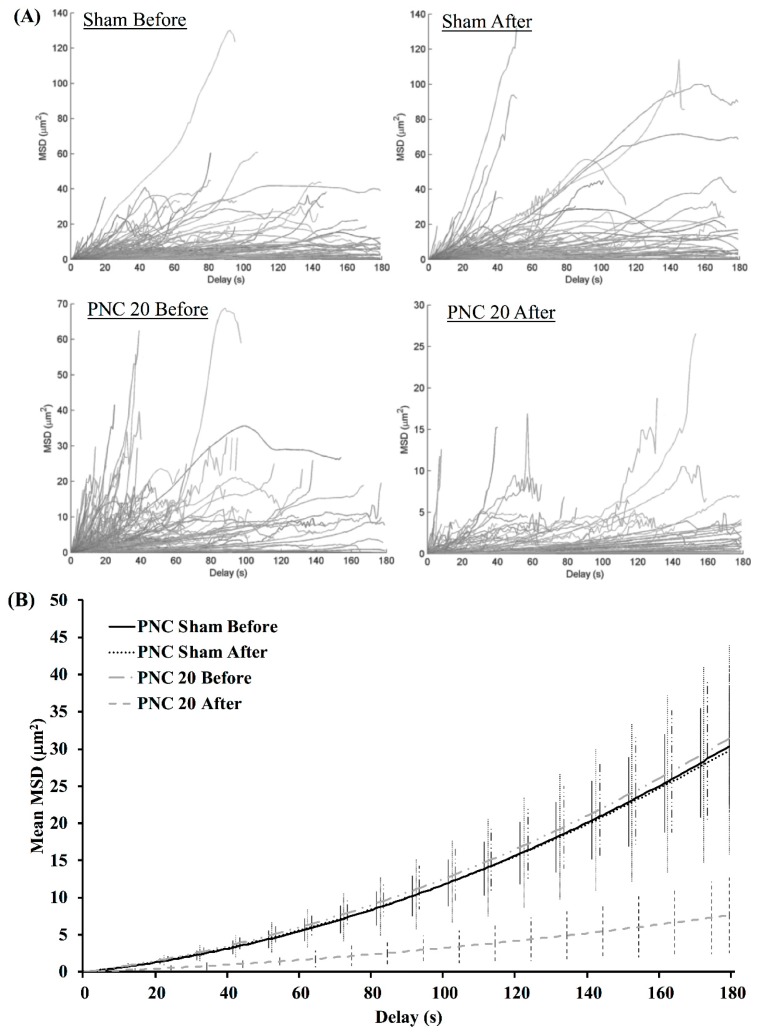
(**A**) Representative directed motion mean square displacement (MSD) plots for a sham-exposed cell (**top**) and a cell bathed in PEG-containing solution and exposed to 20 nsPEF (**bottom**); before (**left**) and after (**right**) exposures. (**B**) Corresponding mean MSD values plotted using the median coefficients of the polynomial fits are shown. Error bars represent one standard deviation from the mean.

Representative MSD values calculated for all particular tracks within a single cell are shown in Figure 3A, for sham and 20 pulse exposures in PEG-containing solution without Ca^2+^ (PNC). The apparent range of MSD values remains the same for the sham condition; however, following the nsPEF exposures, the range decreases and many of the tracks that persist the entire 180 s have MSD values less than 5 μm^2^. The median coefficients from polynomial fits of the mean MSD values per cell are plotted in Figure 3B, showing similar values before exposure for sham and PNC 20. After exposure, the MSD values for PNC 20 are reduced.

**Figure 4:**
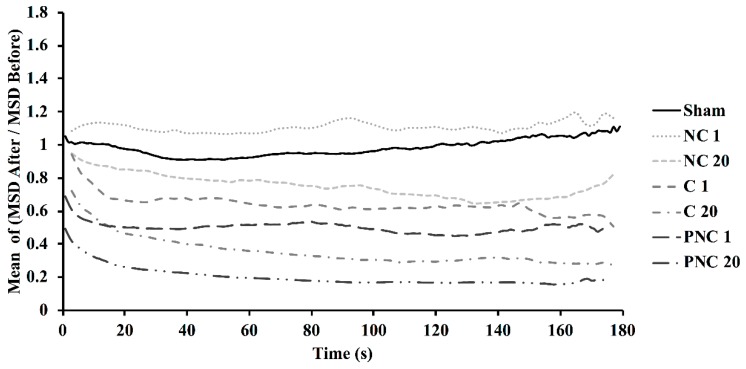
Ratios of the pre- and post-nsPEF means of cumulative MSD over 180 s for all lysosome tracks in cells in the presence or absence of external Ca^2+^, or in the presence of PEG without Ca^2+^, are shown. Exposures consisted of 1 or 20, 600-ns duration pulses at 16.2 kV/cm, or a sham exposure.

Whereas the representative conditions in Figure 3 have been chosen for high contrast, Figure 4 shows relative MSD values, i.e., the cell-to-cell mean of the ratios of MSD after to before, over time, for each condition tested. The ratio for the sham remains around unity. A single pulse in Ca^2+^-depleted solution (NC 1) elicits an increased MSD compared to before the exposure. All other conditions (i.e., extracellular Ca^2+^, extracellular PEG, or 20 pulses) result in suppressed MSD values following exposure, with PNC 20 having the greatest decrease. The relative MSD values for the PC 1 and PC 20 conditions are shown in Figure S7.

**Figure 5:**
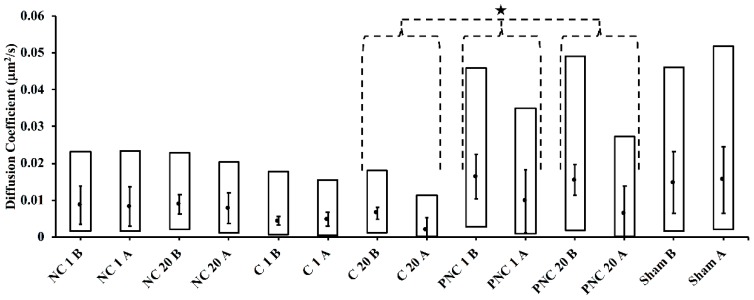
Diffusion coefficients for each condition tested are shown before and after exposure in box plots. The upper limit of the box represents the third quartile, while the bottom limit is the first quartile. The median is given as the middle dot. The star shape (★) denotes a statistically significant difference according to the K-S test at alpha = 0.01 compared to pre-exposure. Error bars represent one standard deviation from the cell-to-cell mean value.

By fitting the MSD curve for each track with the parabolic expression (Equation (1)), both the diffusion coefficient and particle velocity are yielded [7,16]. The diffusion coefficients for each condition, before and after exposure (B and A, respectively), are represented in the box plots in Figure 5. Diffusion coefficients decrease following exposure by a statistically significant amount for C 20, PNC 1, and PNC 20, according to the K-S test at alpha = 0.01. Diffusion coefficients are higher, although not significantly, for cells within PEG-containing solution.

**Figure 6:**
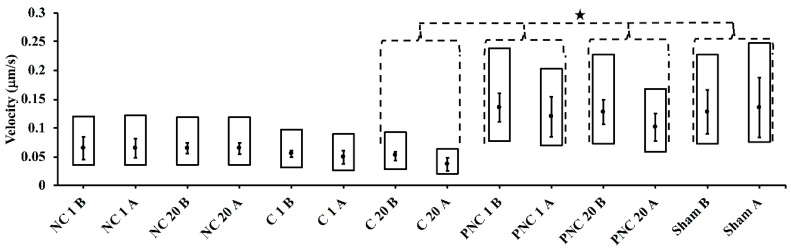
Track velocities for each condition tested are shown before and after exposure in box plots. The star shape (★) denotes a statistically significant difference according to the K-S test at alpha = 0.01 compared to pre-exposure. Error bars represent one standard deviation from the cell-to-cell mean value.

Resultant particle velocities (measured frame-to-frame) for each condition are represented in the box plots in Figure 6. Velocities decrease following nsPEF exposure by a statistically significant amount for C 20, PNC 1, and PNC 20 according to the K-S test at alpha = 0.01. For the sham condition, velocities significantly increased during the second half of image acquisition (181–360 s). The cell-to-cell mean velocity for cells within PEG-containing solutions are significantly greater than the mean velocities found in cells within the other solutions, according to a two-tailed Student’s *t*-test at alpha = 0.01. The diffusion coefficients and velocities for the PC 1 and PC 20 conditions are given in Table S1.

**Figure 7:**
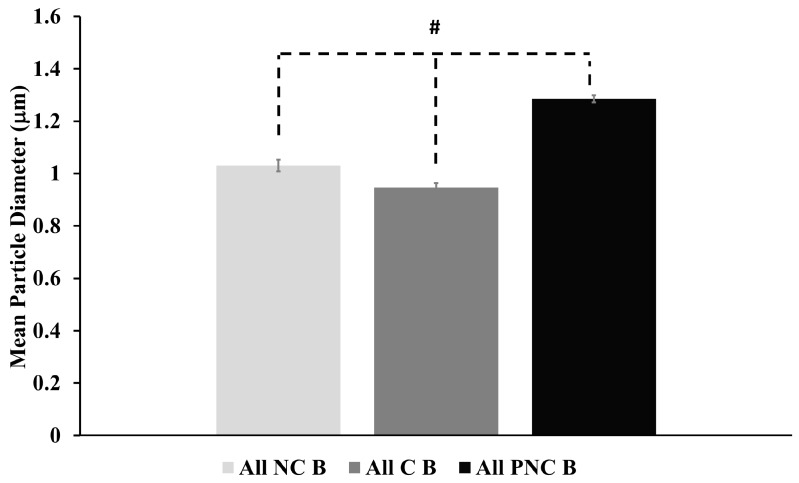
Mean diameters of tracked particles for each type of solution (C, NC, and PNC) are shown. The hashtag shape (#) denotes a statistically significant difference according to Student’s two-tailed *t*-tests at alpha = 0.01. Error bars represent one standard deviation from the cell-to-cell mean value.

The cell-to-cell mean diameters of tracked particles before exposure are shown in Figure 7. The diameters did not change significantly following nsPEF exposure in any condition. However, each type of solution exhibited significantly different diameters, according to Student’s two-tailed *t*-tests at alpha = 0.01, as denoted by the symbol (#). In standard solution (C), the mean diameter is 0.947 ± 0.0575 μm. In Ca^2+^-free solution (NC), the mean diameter is 1.030 ± 0.0711 μm. In PEG-containing solution (PNC), the mean diameter is 1.29 ± 0.0845 μm.

## 4. Discussion

The purpose of this study is to quantify the effect of nsPEF exposure on intracellular lysosome translocation. An SPT method is used to perform an MSD analysis of lysosome movement before and after nsPEF exposure. The tracking and MSD analysis routines herein have been used in multiple publications [14,15], including for analyses of active intracellular transport of proteins associated with cytoskeletal structures [18,19,20]. A nonlinear (i.e., parabolic) fit of MSD curves indicates the single particle is undergoing directed motion [7,16]. This study quantitatively shows that nsPEF exposure impacts lysosome motion in a manner partly dependent upon intracellular Ca^2+^ concentrations and colloid osmotic balance. Intracellular lysosome movement is inhibited by nsPEF exposure(s) in the presence of PEG 300-containing solution or in the presence of extracellular Ca^2+^.

The presence or absence of extracellular Ca^2+^, and the presence of extracellular PEG during exposures, are used to begin evaluating the underpinning mechanism(s) of lysosome stagnation. The number of tracks containing at least five consecutive detected spots and undergoing directed motion decreases by an observable (Figure 1) and statistically significant amount (Figure 2) upon nsPEF exposure to 20 pulses, in solution with Ca^2+^, or in solution with PEG. This confirms earlier published observations that intracellular translocation of lysosomes appears to cease upon perfusion of Ca^2+^-containing solution over a cell exposed to nsPEF only 30 s before [11]. An increased, localized concentration of Ca^2+^ initiates a host of pathways and changes the association of biomolecules with cytoskeletal filaments, and PEG 300 is known to suppress the rate of cell swelling following exposure to 600 ns PEF [11]. PEG 300 can cause a small amount of cell shrinkage approximately within the first 5 minutes after nsPEF exposure [9]. Given that lysosome motion is inhibited by switching between two microtubules at intersections that have separations less than 100 nm [21], and that cell shrinkage likely constricts intracellular volumes and decreases microtubule separations, it is possible that a colloid osmotic imbalance contributes to the nsPEF-exposure-dependent, significant decreases in the number of tracks, diffusion coefficients, and particle velocities in the presence of PEG 300. Without Ca^2+^ or PEG in solution, an appreciable percent decrease in tracks still occurs, indicating that influx of extracellular Ca^2+^ or changes in cell volume are not the only factors affecting the mechanisms of lysosome movement upon nsPEF exposure.

While intracellular lysosome translocation depends on localized Ca^2+^ concentrations [13], and nsPEF exposure(s) can cause an influx of extracellular Ca^2+^ and release of intracellular Ca^2+^ stores [22], the effect of nsPEF exposure on lysosome translocation is not shown to entirely depend on changes of localized Ca^2+^ concentrations. Multiple nsPEF exposures in the absence of both PEG and extracellular Ca^2+^ elicit significant decreases in the number of tracks and an apparent decrease in the average MSD. This effect on only the number of tracks and MSs (yet, without significant change in diffusion coefficient or velocity) could be an artifact related to changes in cell volumes, given that the thickness of the confocal imaging plane was only 2 μm, and three-dimensional (3-D) particle tracking was not achievable. The 2-μm focal thickness is approximately twice the mean particle diameters tracked, which allows for capture of a lysosome rotating about a single microtubule filament. However, tracking of 3-D movement is precluded by these experimental methods, and, as mentioned in one of our prior publications [11], significant dimensional changes of cells following nsPEF exposure could shift the confocal imaging plane. This ‘loss’ of particle tracks due to significant changes in cell volume is mitigated by limiting the analyses of tracks to those that are identified to contain spots in more than four consecutive time points, according to the tracking parameters described in Materials and Methods. Although this means that the decrease in number of tracks and MSD could still be attributed to changes in cell volume, overall changes in the diffusion coefficient and velocity are impacted much less by displacement of particle tracks out of the focal plane.

Diffusion coefficients (Figure 5) and velocities (Figure 6) significantly drop following nsPEF exposure in solutions containing PEG (PNC 1 and PNC 20) or Ca^2+^ (C 20). Values of diffusion coefficients calculated by the nonlinear analysis of lysosome motions are consistent with published ranges of 0.003–0.07 μm^2^/s [23,24]. Particle velocities determined by frame-to-frame displacement of lysosomes fall slightly below published ranges of approximately 0.2–0.7 μm/s for average directed motion velocities in untreated or control cells [21,23,24,25]. However, the median velocities are faster than the average speed of 0.026 ± 0.002 μm/s reported by Cordonnier et al. [25] for random motion of lysosomes. The disparity among values in different solutions and in comparison to published results could be partly attributed to different imaging conditions (e.g., solutions, frame rates, particle density, and particle size [26]), specific cell cultures, and image-processing and analysis protocols. As mentioned in the Materials and Methods section above, image sequences have been acquired at a frame rate of 3 frame/s for a total of 360 s in solutions without PEG, and at a frame rate of 1 frame/s for a total of 480 s in solutions with PEG. Importantly, the relative values before and after exposure consistently demonstrate the impact of nsPEF on lysosomal translocation.

The volume of lysosomes is expected to influence translocation if motion depends upon the microtubule network. The differences in mean tracked particle diameters for each type of solution correlate with differences in diffusion coefficients and velocities. The larger particles exhibit faster passive and active transport. This relationship between particle size and motion contrasts with the results of Bandyopadhyay et al. [23]. They report that lysosomes, osmotically swollen in BS-C-1 monkey kidney epithelial cells in the presence of a 50 mM sucrose solution to a median diameter of 1.3 μm, exhibit significantly decreased MSD values and diffusion coefficients, and yet no significant difference in velocity distributions according to a K-S test. This makes sense if interfilament spacing of the microtubule network remains unchanged. Exposure to nsPEF in PEG 300 leads to a colloid osmotic imbalance and slight shrinking of cells, which likely diminishes the spacing between microtubule filaments. Otherwise, 20 pulses of 600 ns PEF in Ca^2+^-containing solution induces microtubule collapse [11,27]. For both conditions, some lysosomal motion can be postulated to switch from directed to confined; and MSD, diffusion and velocity decrease.

Herein, the mean diameters of tracked particles (Figure 7) approximately double the accepted values of 50–500 nm for lysosome diameters. The spatial resolution of fluorescence microscopy depends on the optical diffraction limit of about 300 nm; and so, the size of the tracked particles may not reflect the actual size of lysosomes. Regardless, intracellular vesicles of hundreds of nm in diameter potentially could be porated by consecutive nsPEF exposures [28], inhibiting intracellular movement; yet, such a supposition requires further exploration. It is also possible that the compartments being analyzed are autolysosomes, with expected diameters of 500 nm to about 1000 nm [29]. However, autolysosomes amass near the perinuclear region [30], which is not observed consistently in the cells studied here (Figure 1). Previous work from this lab shows that exposure to multiple pulses of 10 ns PEF (150 kV/cm) activates autophagy in U937 and CHO-K1 cells [31]. Inhibition of intracellular lysosome translocation precludes autophagic repair, which, as Ullery et al. point out [31], could help explain nsPEF dose-dependent cell death mechanisms.

The connections shown here and elsewhere [11] among particle size, cytoskeleton integrity, and intracellular translocation warrant elucidation of the mechanisms by which lysosomes and the cytoskeleton network respond to nsPEF exposure. This could play a role in the application of nsPEF to medical treatments, e.g., of cancer in which specific cell types are targeted for ablation [27,32]. Future efforts should distinguish between lysosomes and autolysosomes, improve the accuracy of tracking methods (e.g., using targeted quantum dots), elucidate the role of cortical actin in membrane repair following nsPEF [33], and begin to elucidate any role of lysosomal ion channels or pore formation within lysosomes in cellular responses to nsPEF exposure.

## Supplementary Materials

The following are available online at http://www.mdpi.com/2306-5354/5/4/103/s1, Figure S1: Representative oscilloscope trace of a single 600 ns electric pulse with peak amplitude of about −460 V delivered across parallel tungsten rod microelectrodes in solution with PEG 300 and without Ca^2+^ (PNC); Figure S2: Particle tracks of lysosomes within a CHO cell over 180 s (a) before and (b) after exposure to a single 600 ns PEF of 16.2 kV/cm in solution without Ca^2+^ (NC1). Black circles (•) represent initial particle spots, while red squares (**■**) indicate final spot detections. Tracks are represented by arbitrarily colored lines; Figure S3: Particle tracks of lysosomes within a CHO cell over 180 s (a) before and (b) after exposure to a single 600 ns PEF of 16.2 kV/cm in solution with Ca^2+^ (C1). Black circles (•) represent initial particle spots, while red squares (**■**) indicate final spot detections. Tracks are represented by arbitrarily colored lines; Figure S4: Particle tracks of lysosomes within a CHO cell over 180 s (a) before and (b) after exposure to a single 600 ns PEF of 16.2 kV/cm in solution with PEG 300 and without Ca^2+^ (PNC1). Black circles (•) represent initial particle spots, while red squares (**■**) indicate final spot detections. Tracks are represented by arbitrarily colored lines; Figure S5: Particle tracks of lysosomes within a CHO cell over 180 s (a) before and (b) after a sham exposure (i.e., no exposure) in solution with PEG 300 and without Ca^2+^ (PNC1). Black circles (•) represent initial particle spots, while red squares (**■**) indicate final spot detections. Tracks are represented by arbitrarily colored lines. Figure S6: Particle tracks of lysosomes within a CHO cell over 180 s (a) before and (b) after a single exposure in solution with PEG 300 and Ca^2+^ (PC1). Black circles (•) represent initial particle spots, while red squares (**■**) indicate final spot detections. Tracks are represented by arbitrarily colored lines. Figure S7: Average ratios of the mean square displacements for PC 1 and PC 20. Table S1: Results for particles tracked in cells within solution containing extracellular Ca^2+^ and PEG, before and after exposure to 1 (PC 1) or 20 (PC 20) pulses of 600 ns duration at 16.2 kV/cm. Only one cell per type of exposure is represented, and means and standard deviations (std. dev.) are calculated for all particle tracks within the single cell.
