# Supplementary material for: Tracking Lysosome Migration within Chinese Hamster Ovary (CHO) Cells Following Exposure to Nanosecond Pulsed Electric Fields
Source: Bioengineering (Basel). 2018 Nov 23;5(4):103. doi: 10.3390/bioengineering5040103 (PMC6316806; doi:10.3390/bioengineering5040103)
Supplement: Supplementary file 1 [file bioengineering-05-00103-s001.pdf]

bioengineering

Article

# Tracking Lysosome Migration within Chinese Hamster Ovary (CHO) Cells Following Exposure to Nanosecond Pulsed Electric Fields

**Gary L. Thompson [1,*], Hope T. Beier [2] and Bennett L. Ibey [2]**

[1] Department of Chemical Engineering, Rowan University, Glassboro, NJ, USA
[2] Human Effectiveness Directorate, 711th Human Performance Wing, Air Force Research Laboratory, Joint Base San Antonio – Fort Sam Houston, TX, USA
* Correspondence: thompsong@rowan.edu; Tel.: +1-856-256-5357

Received: date; Accepted: date; Published: date

## Supplementary Material

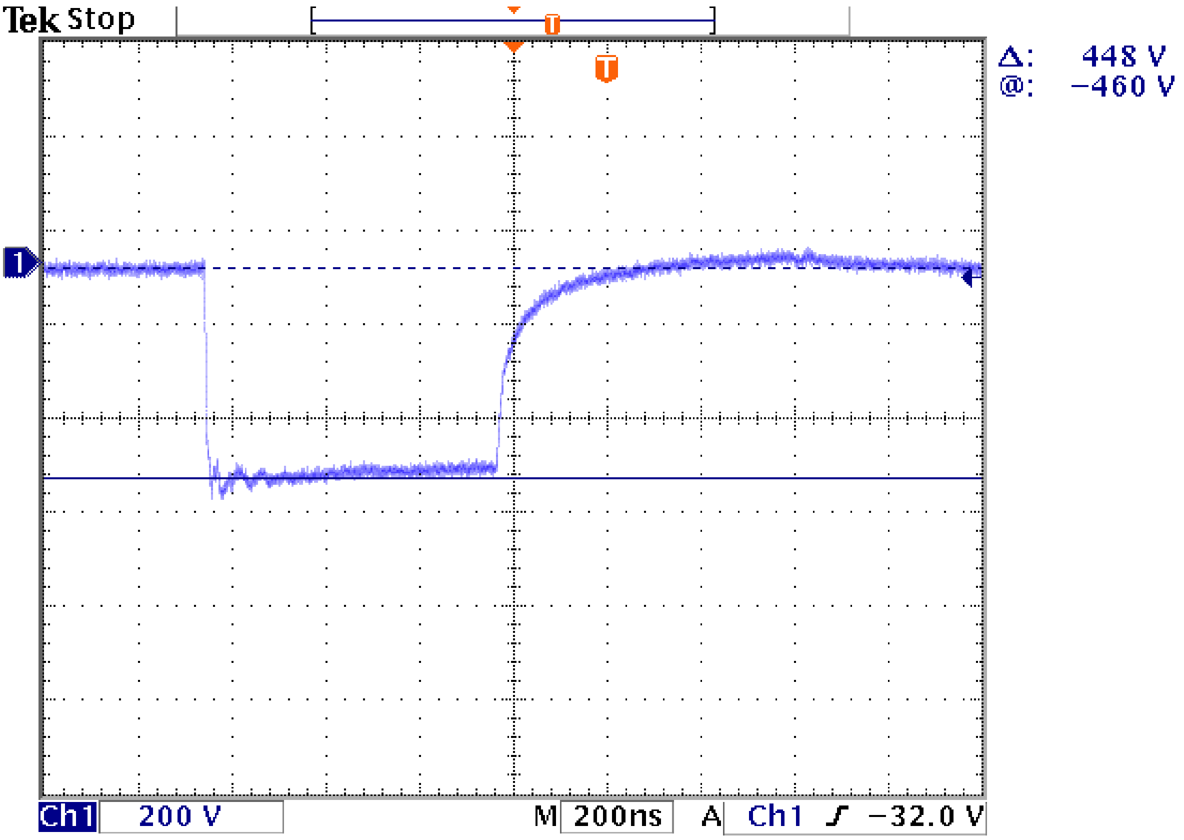


**Figure S1.** Representative oscilloscope trace of a single 600 ns electric pulse with peak amplitude of about -460 V delivered across parallel tungsten rod microelectrodes in solution with PEG 300 and without $Ca^{2+}$ (PNC).

**(a)**

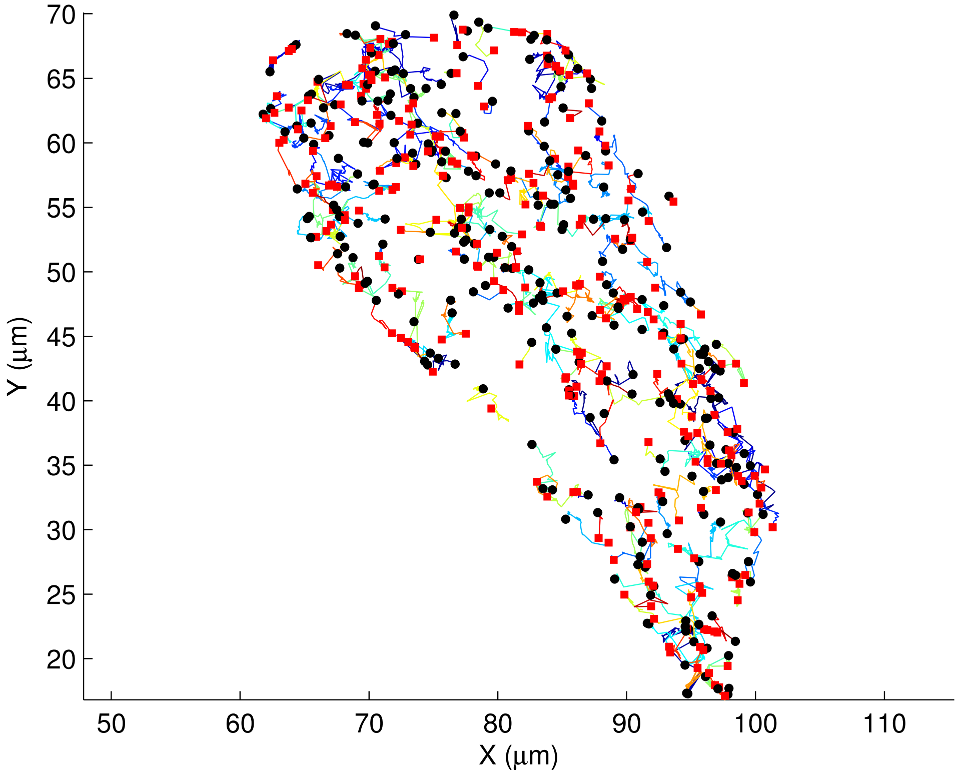


**(b)**

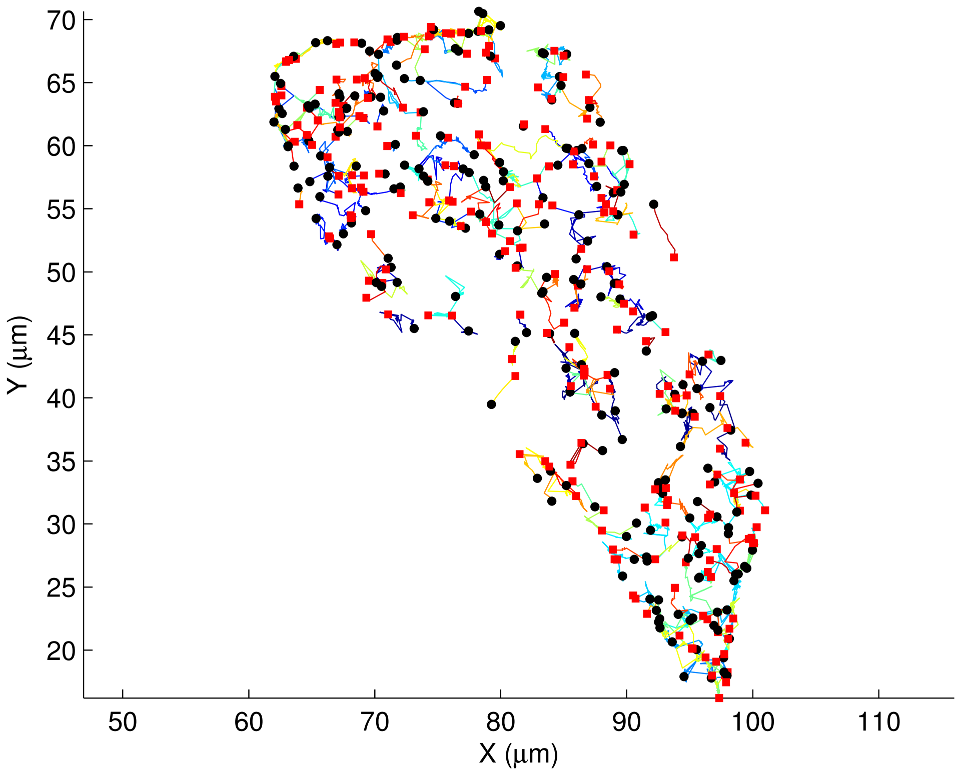


**Figure S2.** Particle tracks of lysosomes within a CHO cell over 180 s (a) before and (b) after exposure to a single 600 ns PEF of 16.2 kV/cm in solution without $Ca^{2+}$ (NC1). Black circles (•) represent initial particle spots, while red squares (■) indicate final spot detections. Tracks are represented by arbitrarily colored lines.

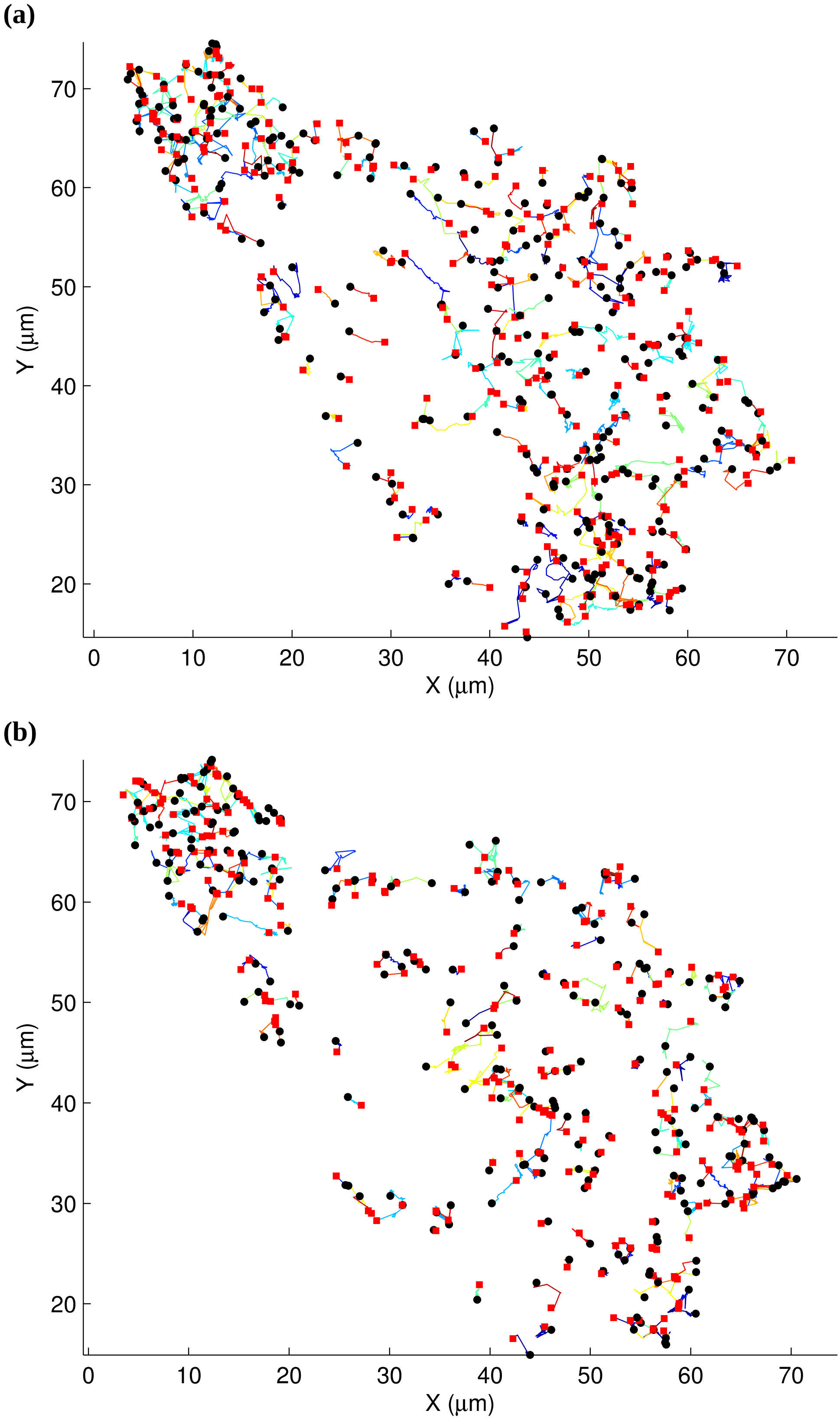


**Figure S3.** Particle tracks of lysosomes within a CHO cell over 180 s (a) before and (b) after exposure to a single 600 ns PEF of 16.2 kV/cm in solution with $Ca^{2+}$ (C1). Black circles (•) represent initial particle spots, while red squares (■) indicate final spot detections. Tracks are represented by arbitrarily colored lines.

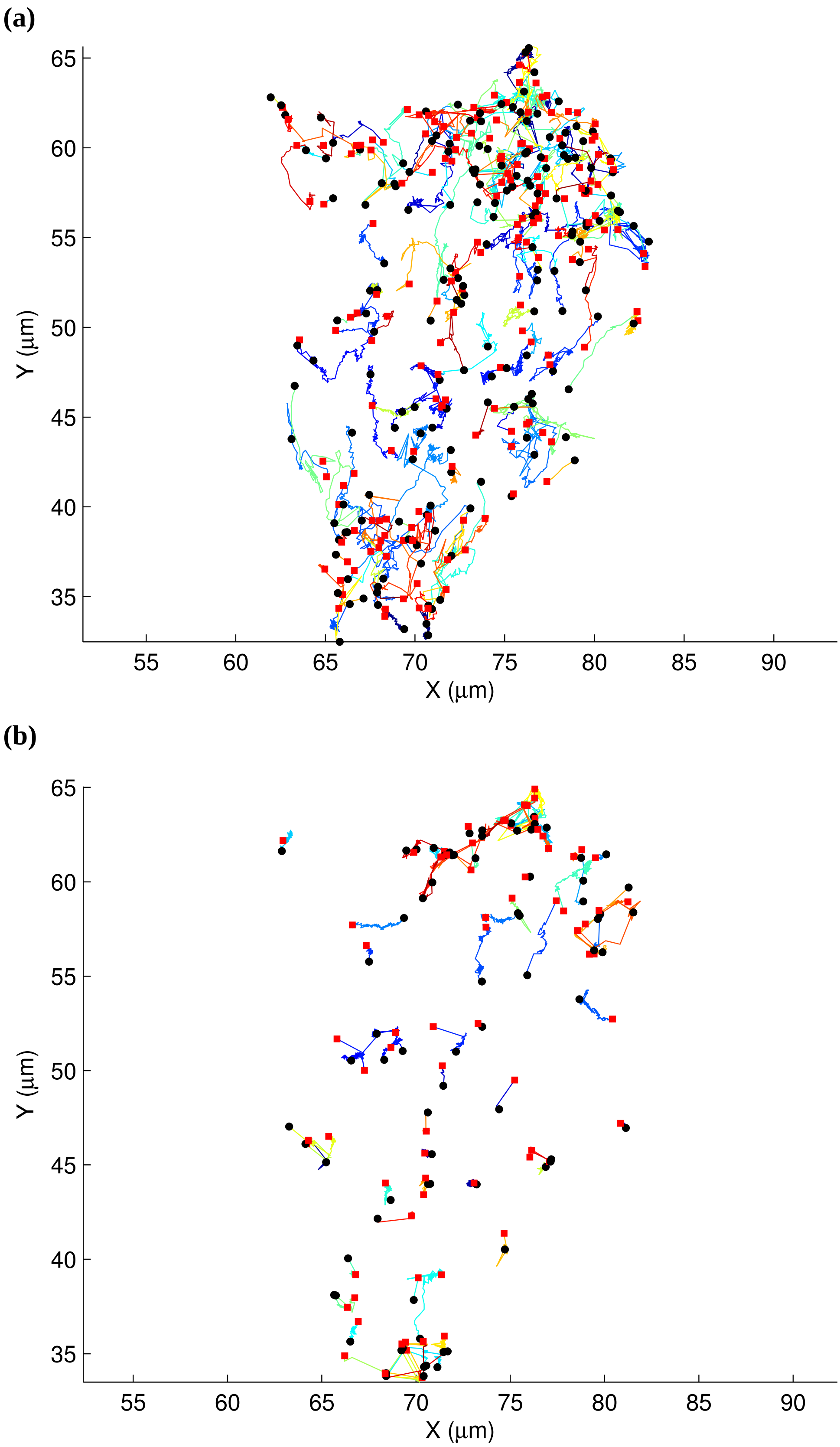


**Figure S4.** Particle tracks of lysosomes within a CHO cell over 180 s (a) before and (b) after exposure to a single 600 ns PEF of 16.2 kV/cm in solution with PEG 300 and without $Ca^{2+}$ (PNC1). Black circles (•) represent initial particle spots, while red squares (■) indicate final spot detections. Tracks are represented by arbitrarily colored lines.

**(a)**

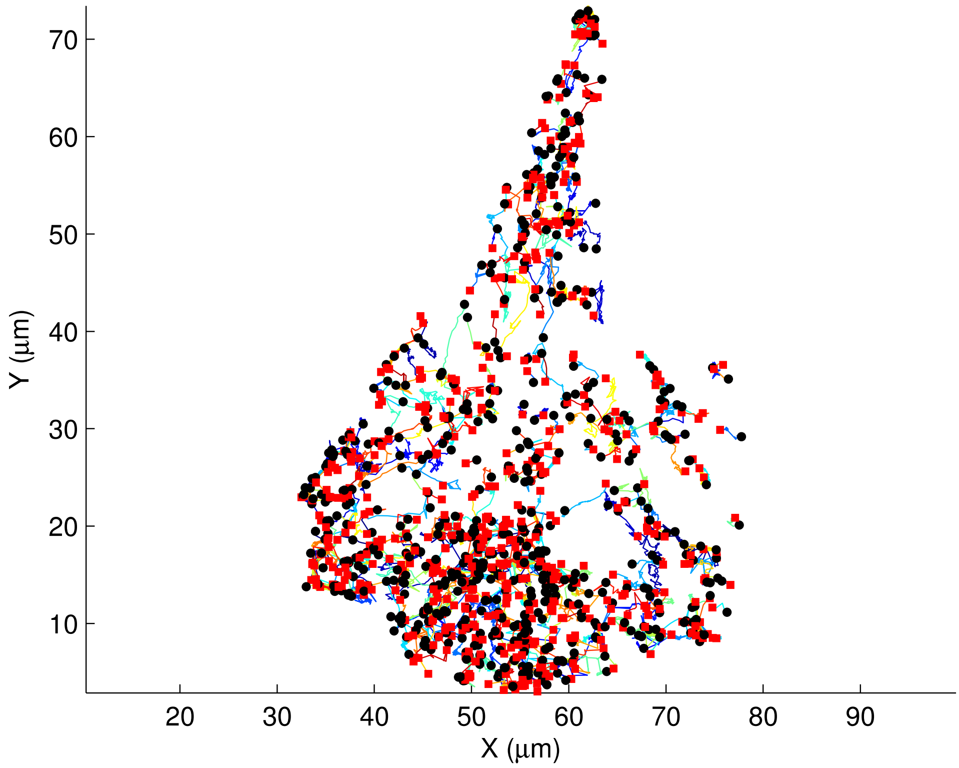


**(b)**

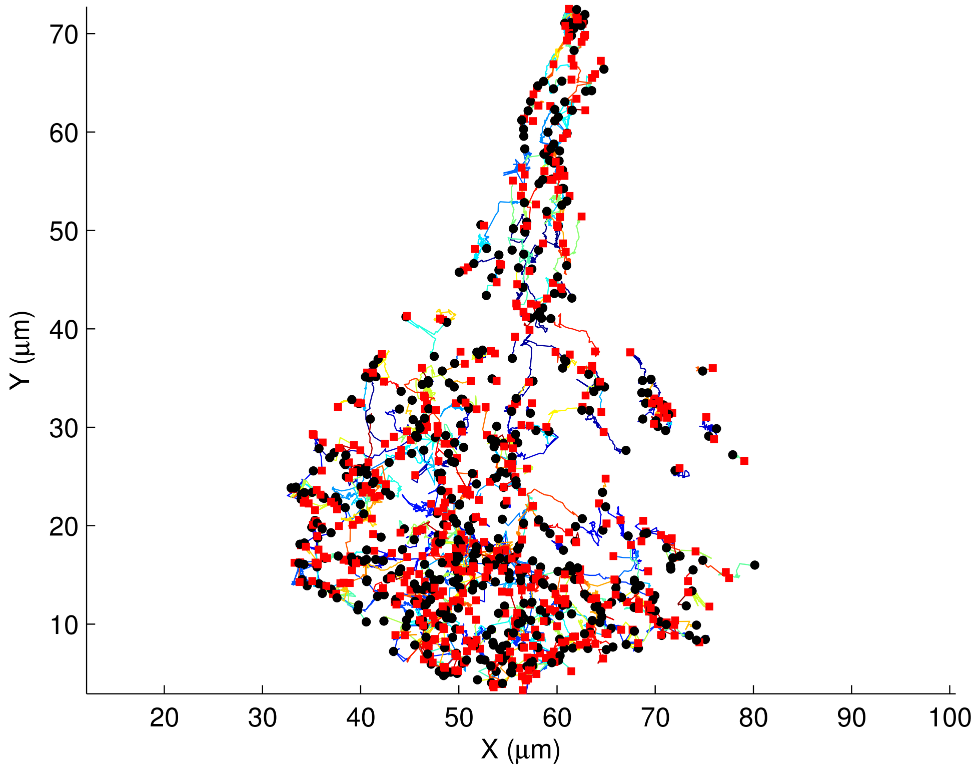


**Figure S5.** Particle tracks of lysosomes within a CHO cell over 180 s (a) before and (b) after a sham exposure (i.e. no exposure) in solution with PEG 300 and without $Ca^{2+}$ (PNC1). Black circles (•) represent initial particle spots, while red squares (■) indicate final spot detections. Tracks are represented by arbitrarily colored lines.

**(a)**

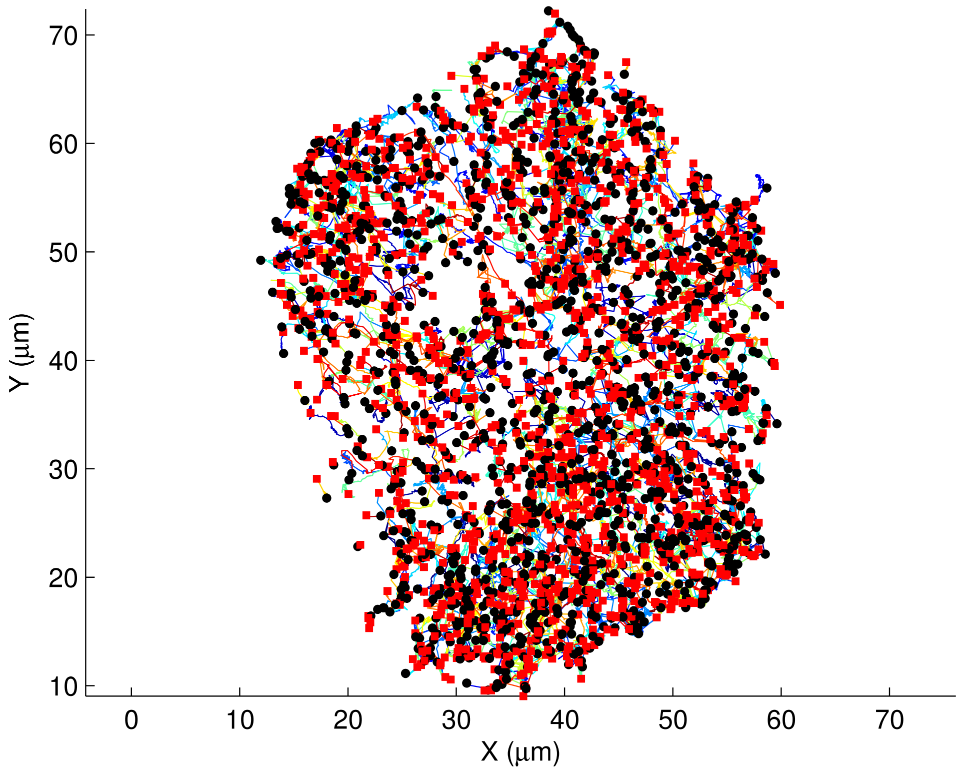


**(b)**

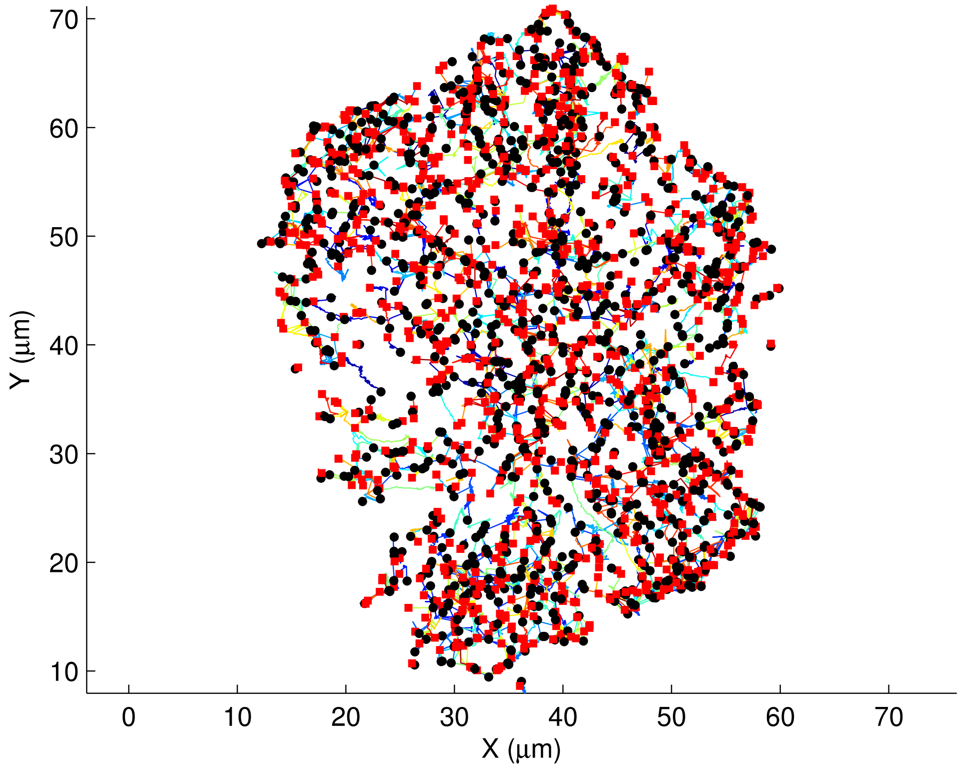


**Figure S6.** Particle tracks of lysosomes within a CHO cell over 180 s (a) before and (b) after a sham exposure (i.e. no exposure) in solution with PEG 300 and with $Ca^{2+}$ (PC1). Black circles (●) represent initial particle spots, while red squares (■) indicate final spot detections. Tracks are represented by arbitrarily colored lines.

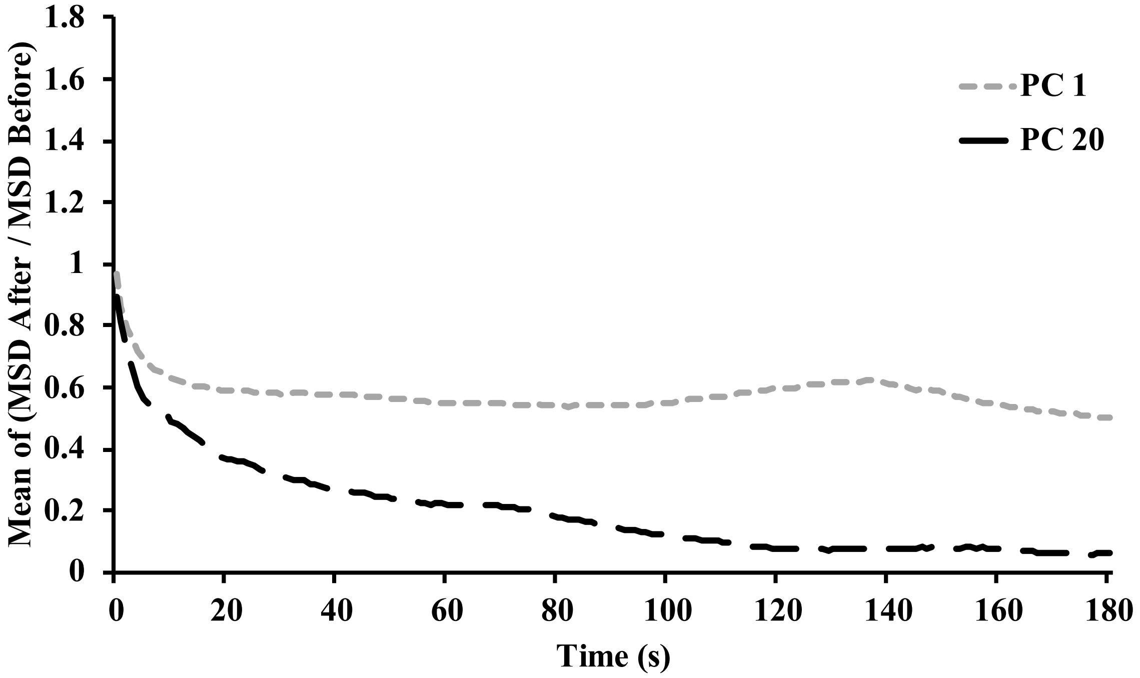


**Figure S7.** Ratios of the pre- and post-nsPEF means of cumulative MSD over 180 s for all lysosome tracks in cells in the presence extracellular $Ca^{2+}$ and in the presence of PEG 300 are shown. Only a single cell is analyzed for each condition. Exposures consisted of 1 or 20, 600-ns duration pulses at 16.2 kV/cm.

**Table S1.** Results for particles tracked in cells within solution containing extracellular $Ca^{2+}$ and PEG, before and after exposure to 1 (PC 1) or 20 (PC 20) pulses of 600 ns duration at 16.2 kV/cm. Only one cell per type of exposure is represented, and means and standard deviations (std. dev.) are calculated for all particle tracks within the single cell.

| | **PC 1** | **PC 20** |
|---|---|---|
| Diffusion Coefficient, Before, Mean ± Std. Dev. ($\mu m^2/s$) | 0.0340 ± 0.0578 | 0.0302 ± 0.0786 |
| Diffusion Coefficient, After, Mean ± Std. Dev. ($\mu m^2/s$) | 0.0295 ± 0.0556 | 0.0331 ± 0.0745 |
| Velocity, Before, Mean ± Std. Dev. (μm/s) | 0.214 ± 0.283 | 0.165 ± 0.242 |
| Velocity, After, Mean ± Std. Dev. (μm/s) | 0.200 ± 0.286 | 0.135 ± 0.238 |
| % Decrease in Number of Particle Tracks | 26.1 | 54.6 |

© 2018 by the authors. Licensee MDPI, Basel, Switzerland. This article is an open access article distributed under the terms and conditions of the Creative Commons Attribution (CC BY) license (http://creativecommons.org/licenses/by/4.0/).
